# Development and biological evaluation of Ti6Al7Nb scaffold implants coated with gentamycin-saturated bacterial cellulose biomaterial

**DOI:** 10.1371/journal.pone.0205205

**Published:** 2018-10-24

**Authors:** Karolina Dydak, Adam Junka, Patrycja Szymczyk, Grzegorz Chodaczek, Monika Toporkiewicz, Karol Fijałkowski, Bartłomiej Dudek, Marzenna Bartoszewicz

**Affiliations:** 1 Department of Pharmaceutical Microbiology and Parasitology, Wroclaw Medical University, Wrocław, Poland; 2 Center for Advanced Manufacturing Technologies (CAMT/FPC), Faculty of Mechanical Engineering, Wroclaw University of Science and Technology, Wrocław, Poland; 3 Laboratory of Confocal Microscopy, Polish Center for Technology Development PORT, Wrocław, Wrocław, Poland; 4 Department of Immunology, Microbiology and Physiological Chemistry, Faculty of Biotechnology and Animal Husbandry, West Pomeranian University of Technology, Szczecin, Szczecin, Poland; 5 Laboratory of Microbiology, Polish Center for Technology Development PORT, Wrocław, Wrocław, Poland; VIT University, INDIA

## Abstract

Herein we present an innovative method of coating the surface of Titanium-Aluminium-Niobium bone scaffold implants with bacterial cellulose (BC) polymer saturated with antibiotic. Customized Ti6Al7Nb scaffolds manufactured using Selective Laser Melting were immersed in a suspension of *Komagataeibacter xylinus* bacteria which displays an ability to produce a 3-dimensional structure of bio-cellulose polymer. The process of complete implant coating with BC took on average 7 days. Subsequently, the BC matrix was cleansed by means of alkaline lysis and saturated with gentamycin. Scanning electron microscopy revealed that BC adheres and penetrates into the implant scaffold structure. The viability and development of the cellular layer on BC micro-structure were visualized by means of confocal microscopy. The BC-coated implants displayed a significantly lower cytotoxicity against osteoblast and fibroblast cell cultures *in vitro* in comparison to non-coated implants. It was also noted that gentamycin released from BC-coated implants inhibited the growth of *Staphylococcus aureus* cultures *in vitro*, confirming the suitability of such implant modification for preventing hostile microbial colonization. As demonstrated using digital microscopy, the procedure used for implant coating and BC chemical cleansing did not flaw the biomaterial structure. The results presented herein are of high translational value with regard to future use of customized, BC-coated and antibiotic-saturated implants designed for use in orthopedic applications to speed up recovery and to reduce the risk of musculoskeletal infections.

## Introduction

A perfect bone implant should be light, mechanically durable, resistant to chemicals and corrosion, as well as non-toxic, bio-compatible and protected from microbial contamination by surface modification [1–4]. Implants composed of titanium, aluminum and vanadium alloy (Ti6Al4V) are examples of modern orthopedic products, broadly used in contemporary orthopedics. Implants containing the above alloy display not only high strength and fracture toughness, but also high resistance to corrosion. However, it should be noted that vanadium or its oxide (V_2_O_5_) content may have allergic or cytotoxic potential. Therefore, a new generation of biomaterials, devoid of vanadium, has been developed and gained high interest of researchers and scientists working in the orthopedic-related field. The new generation implants made of titanium, aluminum and niobium alloy (Ti6Al7Nb) are characterized not only by high strength, low modulus and low density. They are also resistant to mechanical and biological corrosion, are characterized by high biocompatibility and low cytotoxicity [5–7].

It is widely accepted that bone implants should contain pores and have a scaffold structure. Such properties contribute to a higher ratio of adhesion and proliferation of eukaryotic bone cells referred to as osteoblasts [8]. The above results in a higher speed of implant incorporation into the bone tissue and recovery in a clinical setting. In this context, we have already demonstrated the suitability of Selective Laser Melting (SLM) for fabrication of such Ti6AL7Nb complex scaffolds as the ones used in the current study [9,7].

Bone implants should also display high biocompatibility which can be achieved or increased by e.g. implant surface modification with a bio-polymeric layer. Such implant surface modifications have a beneficial impact on the interactions between the implant and the immune system and therefore decrease the risk of implant rejection [8].

Bacterial cellulose (BC) produced by *Komagataeibacter xylinus* may be a promising alternative to currently used bio-polymers applied for implant surface modification. Bacterial cellulose displays high crystallinity and polymerization ratio. Moreover, it is devoid of such impurities as hemicelluloses or lignin. BC is also devoid of cytotoxic activity and it is highly compatible with patient tissues and cells [7,10–12]. Implant biocompatibility is of pivotal significance for therapeutic success. However, one should be aware of the significant threat related with the risk of the implant’s microbial contamination. To avoid the above risk, an appropriate antimicrobial should be introduced to the biomaterial structure. In this context, another beneficial BC feature is its high retention ratio. Thanks to this parameter, BC may be saturated with high concentration of anti-inflammatory and antimicrobial drugs, including antibiotics [2–4, 13,14]. Such saturation allows to protect the implant and the surrounding bone from microbial contamination and subsequent biofilm formation. It has already been proven that the release of drugs from BC is of continuous, pro-longed nature [15,16] which is a mandatory feature with regard to antimicrobial protection of the bone. The release of drugs from BC may be additionally prolonged by exposing the cellulose-producing bacteria to Rotating Magnetic Field [17] or by modifying the composition of culturing media [18].

Gentamycin is one of the drugs most commonly used in the treatment of bone infection. This antibiotic also serves as an active substance of many products designed for use in bone recovery (hydroxyapatite cements, gentamycin-collagen sponges, etc.). Its antimicrobial spectrum includes the most common etiological factors of peri-implant infections, including *Staphylococcus aureus* [19]. Moreover, it seems that at least at some concentrations, gentamycin does not deteriorate osteoblast proliferation [20,21].

Therefore, the aim of the present work was to combine the favorable physical parameters of the Ti6Al7Nb alloy with high bio-compatibility and high retention ratio of BC and with antimicrobial properties of gentamycin in order to fabricate a scaffold bone implant of highly desirable properties and to test it in an *in vitro* setting.

## Materials and methods

### Strain and culture conditions

To obtain bacterial cellulose, *K*. *xylinus* ATCC 5352 strain was used. Standard Hestrin-Schramm (H-S) medium and stable H-S agar medium [22] (all compounds from Stanlab, Poland) were applied for strain culturing. Incubation temperature was 28°C (incubator INCU-Line IL 115, VWR, Germany).

The reference ATCC opportunistic pathogen, namely *S*. *aureus* 6538 was used to test the antimicrobial activity of gentamycin released from BC-coated implant. For *S*. *aureus* culturing, the applied liquid medium was Tryptic Soy Broth (Sigma-Aldrich, Germany), while culturing on solid media was performed using Columbia Agar (Sigma-Aldrich, Germany) and in the case of procedures described in the section “Antimicrobial properties of BC-coated implants saturated with gentamycin”—Müller-Hinton Agar (Biocorp, Poland).

### Implant manufacturing

The implants were designed and fabricated as described in more detail in our earlier work [7]. Briefly, a model of a porous unit cell built of struts was designed in a cylindrical form (S1 Fig) using computer-aided design (CAD) by means of Magics software (Materialise HQ, Leuven, Belgium). The cylindrical samples with external dimensions of ø 6.2 x 6.0 mm were fabricated using SLM 3-D printer (ReaLizer 50, UK) from Ti6Al7Nb alloy of 20–63 μm particle diameter. Strut diameter was set at 150 μm, while the distance between strut axes was 600 μm. Moreover, we also performed a bigger, cubic scaffold made of Ti6Al4V alloy of 40 to 106 μm particle diameter by means of EBM Arcam A1 (Sweden) device. The cubic model of the specimen used in the study was composed of 48 unit cells that formed a 10 x 10 mm cube. In that case strut diameter was set at 600 μm, while the distance between strut axes was 2500 μm. The results for this type of implant were of supporting nature for the present line of investigation and are placed in Supplementary Information.

### Chemical composition of the scaffolds

In order to determine compliance of the manufactured scaffolds with the requirements of the relevant standards, a chemical composition analysis was carried out. The content of elements such as oxygen, nitrogen, hydrogen and carbon was analyzed. Carbon content was measured by the infrared absorption method (HFIR) after combustion in an induction furnace (18 MHz, 2.2 kW), using CS-600 Leco analyser (LECO Corp., Saint Joseph, MI, US). During the measurement the values of the absorption coefficient of infrared emitted by CO_2_ were analyzed. Oxygen and hydrogen content was measured by infrared (IR) absorption while nitrogen was analyzed by thermal conductivity (TC) performed based on the melt extraction method using the Leco TCH-600 device (LECO Corp., Saint Joseph, MI, US). The results obtained were compared with the binding standard, namely: ASTM F1295-11 (Standard specification for wrought Titanium-6Aluminum-7Niobium alloy for surgical implant applications (UNS R56700).

### Minimal Inhibitory Concentration (MIC) of gentamycin against *Staphylococcus aureus*

MIC assessment was prepared in 96-well titration micro-plates (Corning Life Science, USA). The wells of the plate were filled with 100 μL of TSB medium. Next, 100 μL of 2000 mg/L of gentamycin (MP Biomedicals, USA) was added to the first of the wells and mixed with the medium. Subsequently, geometric dilution of the antibiotic was performed. Next, 100 μL of bacterial suspension (10^5^ cfu/mL) was introduced to each well. The entire plate was incubated at 37°C/24h in a shaker (Schüttler Microplate Shaker, MTS-4, IKA, Germany). The final range of gentamycin concentrations was 0.98 mg/L–500 mg/L. The culture with no antibiotic added served as a positive control, while the sterility control well contained the sterile medium only. After incubation, 5 μL of triphenyl tetrazolium chloride, TTC (Sigma Aldrich, Germany) was added to each well and incubated for 5 h at 37°C. A change of colorless TTC to red formazan confirmed the presence of metabolically active microorganism. The antibiotic concentration in the first colorless well, neighboring to the red well was taken as the MIC value.

### Coating of implants with BC

Previously sterilized by autoclaving for 5 min at 134°C (Vapour Line 135 M, VWR, Germany), the implants were placed in wells of a 24-well plate (Corning Life Science, USA) and immersed in HS medium in such a way that 1–2 mm of their height was above the surface of the medium. Next, 100 μL of *K*. *xylinus* inoculum (of 0.5 MF density measured by densitometer, Erba Lachema s.r.o., Czech Republic) was introduced to the wells. The whole setting was incubated for 48 h at 28°C—until the cellulose membrane was formed on the air-liquid interface. Then, the medium was carefully removed, the implants were aseptically flipped by 180°. As a result, the BC membrane became the basic layer for fresh BC formation and subsequent implant coverage. A very thin layer of the medium was added to the implant-containing well, until the whole implant was covered with BC.

### Cellulose cleansing

The removal of *K*. *xylinus* cells from the cellulose matrix was performed by the standard procedure of alkaline lysis. For this purpose, the implants with a cellulose coating were introduced to 0.1 M NaOH (POCH, Poland) and incubated for 2 h at 80°C. Next, the implants were abundantly rinsed with distilled water until pH stabilized at 7.

### Scanning electron microscopy analysis of BC-coated implants

The implants covered with BC were fixed using 3% glutaraldehyde (POCH, Poland) for 15 min at room temperature. Then, the samples were rinsed twice with phosphate buffer (PBS, Sigma-Aldrich, Germany) to remove the fixative. The next step was dehydration in increasing concentrations of ethanol (25%, 60%, 95%, and 100%; POCH, Poland) for 5 min in each solution. After rinsing off the ethanol, the samples were dried at room temperature. Then, the samples were covered with gold and palladium (60:40; sputter current, 40 mA; sputter time, 50 s) using a Quorum machine (Quorum International, USA) and examined under a Zeiss EVO MA25 scanning electron microscope (Zeiss, Germany).

### Cytotoxicity assay

Neutral Red (NR) cytotoxicity assay was performed in osteoblast (U2-OS, ATCC) and fibroblast (L929, ATCC) cell cultures treated with extracts obtained from the BC membrane-conditioned medium. The extracts were prepared according ISO 10993 standard: Biological evaluation of medical devices; Part 5: Tests for *in vitro* cytotoxicity; Part 12: Biological evaluation of medical devices, sample preparation and reference materials (ISO 10993–5:2009 and ISO/IEC 17025:2005). Briefly, the BC-modified and unmodified implants were introduced to 24-well plate wells filled with the appropriate cell culture media without serum (F12 for osteoblasts or MEM for fibroblasts, both media were purchased from Sigma-Aldrich, Germany) and incubated for 72 h in 5% CO_2_ at 37°C with shaking at 500 rpm (Schüttler Microplate Shaker, MTS-4, IKA, Germany). After incubation, the implants were extruded from the wells and the plates were spin-centrifuged. Next, the resulting supernatants (extracts of 100 μL volume) were introduced to the cell cultures and incubated for 24 h and 48 h in 5% CO_2_ at 37°C. After the specified incubation time, the medium was removed and 100 μL of NR solution (40 μg/mL; Sigma-Aldrich, Germany) was introduced to the wells of the plates. The cells were incubated with NR for 2 h at 37°C. After incubation, the dye was removed, the wells were rinsed with PBS (Sigma-Aldrich, Germany) and left to dry at room temperature. Subsequently, 150 μL of de-stain solution (50% ethanol 96%, 49% deionized water, 1% glacial acetic acid; POCH, Poland) was introduced to each well. The plates were vigorously shaken in a microtiter plate shaker for 30 min until NR was extracted from the cells and formed a homogenous solution. Next, the value of NR absorbance was measured spectrometrically using a microplate reader (Infinite m200, Tecan, Swetzeraland) at 540 nm wavelength. The absorbance value of the cells untreated with the extracts was considered 100% of potential cellular growth (positive control sample).

### Visualization of fibroblasts on BC with confocal microscopy

The cultured L929 fibroblasts were harvested from the flasks with 0.25% Trypsin-EDTA (Thermo Fisher Scientific, USA) and loaded with CellTrace CFSE (Thermo Fisher Scientific, USA) intravital dye according to manufacturer’s instructions. The BC membranes were placed in MEM culture medium in a Petri dish and the labeled L929 cells were transferred on top of the membrane, still immersed in the medium. After 7 days of culture the Petri dish was taken for imaging on an upright Leica SP8 confocal microscope (Leica Microsystems, Germany). Stacks of confocal 8-bit images with a pixel size of 0.728 μm and a 2 μm Z step were acquired using 25× water immersion objective (NA 0.95). The pinhole was set to 1 AU. The cellulose surface was visualized in a reflection mode using a 638 nm laser line. CFSE fluorescence was excited with a 488 nm laser line and 498–568 nm emission range was recorded. The acquisition was performed in a sequential mode. Three-dimensional rendering was performed using Imaris software (Bitplane).

### Impregnation of BC-coated implants with gentamycin

The BC-coated implants cleansed as described in section “Cellulose cleansing” were immersed in 2 mL solutions of gentamycin of 2 μg/mL, 4 μg/mL, 8 μg/mL, 10 μg/mL and 1 mg/mL concentration for 24 h at 4°C. Next, the BC-coated implants were removed from the gentamycin solution, immersed in distilled water (for 10 s) and wiped with filter paper to remove non-absorbed antibiotic. The implants not coated with BC but subjected to saturation with gentamycin served as a control in this experiment.

### Antimicrobial properties of BC-coated implants saturated with gentamycin

The staphylococcal cultures on agar plates were prepared using a method similar to the one used for antibiotic sensitivity estimation (Kirker-Bauer Method), i.e. 0.5 MF solutions of pathogens were swabbed on Müller-Hinton Agar plate. Next, the scaffolds impregnated with 2 μg/mL, 4 μg/mL, 8 μg/mL, 10 μg/mL and 1 mg/mL gentamicin solutions were placed on the agar and subjected to 24 h/37°C incubation. In the next step, bacterial growth inhibition zones around the implants were measured using a ladder. The BC-coated implants with no antibiotic but saline served as a negative control in this experimental setting.

### Cellulose removal from implants

Cellulose was removed from the implants by lysis with cellulase enzyme (Cellsoft, Poland). The process was performed at 37°C at 0.1 M citrate buffer (citric acid and trisodium citrate from POCH, Poland) until complete BC lysis.

### The evaluation of the implant surface after cellulose removal

The evaluation of the implant surface after cellulose removal was performed via microscopic method using a scanning electron microscope (Zeiss EVO MA25, Germany). The aim of the analysis was to examine the scaffolds’ surface topography and to search for potential alterations on the struts surface (scratches, damages, losses) that could arise during the BC coating process.

### Statistical analyses

Calculations were performed using the GraphPad Prism version 7 software. Normality distribution was calculated by means of D’Agostino-Pearson omnibus test. Because all values were non-normally distributed, the Mann-Whitney (rank sum) test was applied. The results of statistical analyses were considered significant if they produced p-values < 0.05.

## Results and discussion

Despite the significant progress of antimicrobial prophylaxis, biomaterial-associated infections (BAIs), including those related with bone implant presence, still pose a significant problem in orthopedics and musculoskeletal traumatology. The tendency for such infection to develop depends on the patient’s general health condition, comorbidities, antimicrobial prophylaxis used, body area/tissue subjected to implantation and, last but definitely not least, on the material and type of implant applied [23]. Implant-related infections may cause non-junction or improper junction of bone fragments, inflammation and spread of microorganisms throughout the patient’s body. They finally result in prolonged hospitalization, decrease in the patient’s life quality and may even lead to death.

Treatment procedures, including surgical debridement of the implants surface and local or systemic antibiotic therapy meant to protect or fight back bacterial presence are often completely ineffective. The above leads to therapeutic failure and obligatory removal of the contaminated implant [23,24]. These challenges are the reason behind numerous efforts to manufacture orthopedic implants which are not only biocompatible but also microbiologically safe.

**Fig 1:**
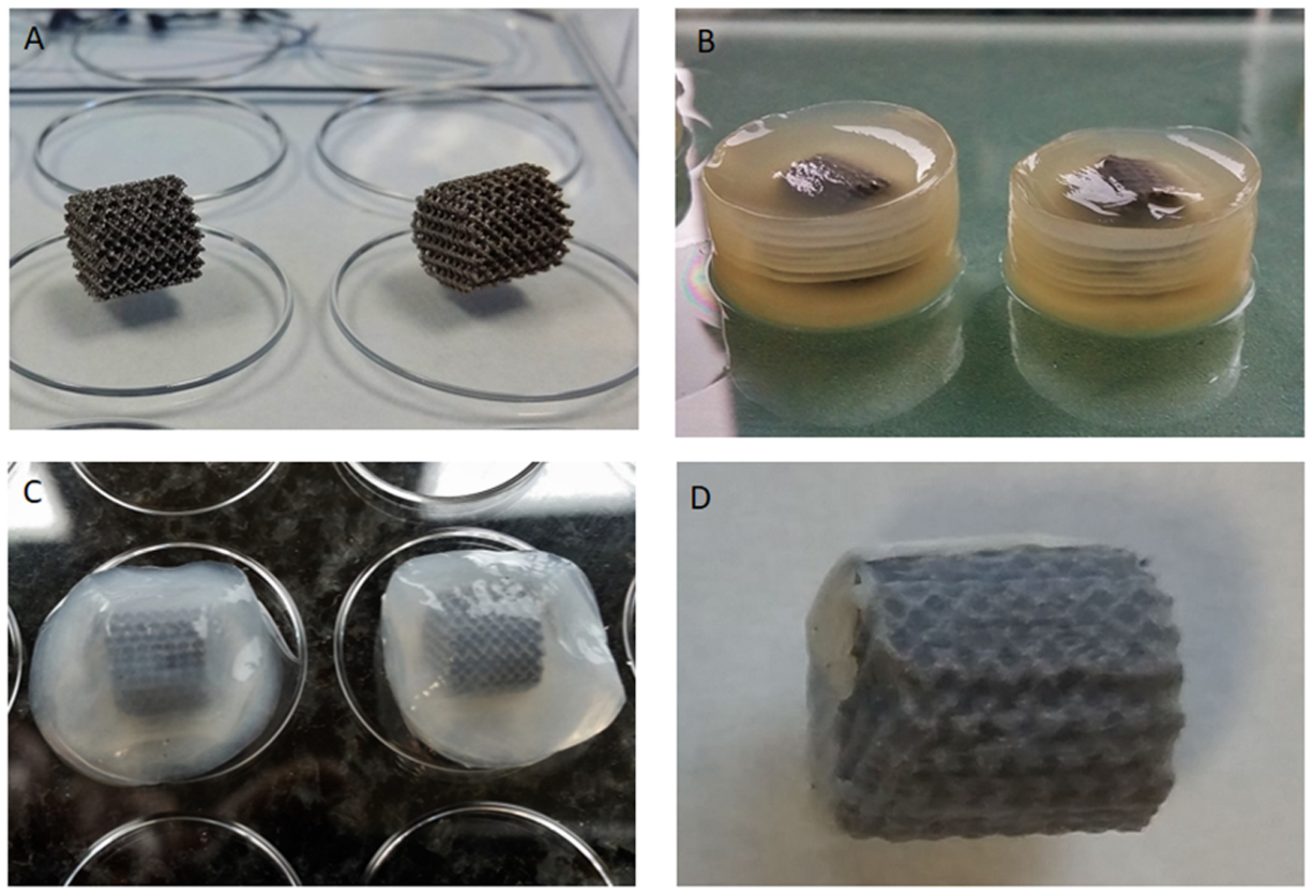
Stages of preparation of BC-coated Ti6Al7Nb scaffold. (A)—native Ti6Al7Nb scaffold; (B)—implant coated with unpurified BC; (C)—BC-coated implant after removal of media leftover and bacteria; (D)—BC-coated Ti6Al7Nb scaffold after partial drying.

Titanium and its alloys are biocompatibile enough to form a functional connection with a living bone tissue in a process called osseointegration [8,25]. Moreover, these biomaterials have lower susceptibility to bacterial adhesion as compared to such other materials used in implantology as latex, polyvinyl chloride, teflon or stainless steel [26,27]. Made of Ti6Al7Nb alloy, the scaffold implants used in the present study display not only good biocompatibility but also relatively low susceptibility to bacterial colonization and subsequent biofilm formation [7,9]. However, we decided to additionally improve Ti6Al7Nb alloy properties by coating it with the most promising biopolymer of recent years, namely bacterial cellulose produced by *K*. *xylinus* bacteria. The main advantages of BC are lack of cytotoxicity and immune response after introduction into the body, high capacity for adsorption of fluids, microfibrillar structure and a simple and relatively inexpensive way of manufacture [10,14,28]. So far, BC has been successfully used in many fields of bio-medical applications [10,29]. In the current study, we simply allowed *K*. *xylinus* bacteria to synthesize BC around and within the Ti6Al7Nb scaffold. The average time required for cellulose to completely cover the cylindrical scaffolds was 7 days. Individual stages of implant coating and cleansing are presented in Fig 1.

**Fig 2:**
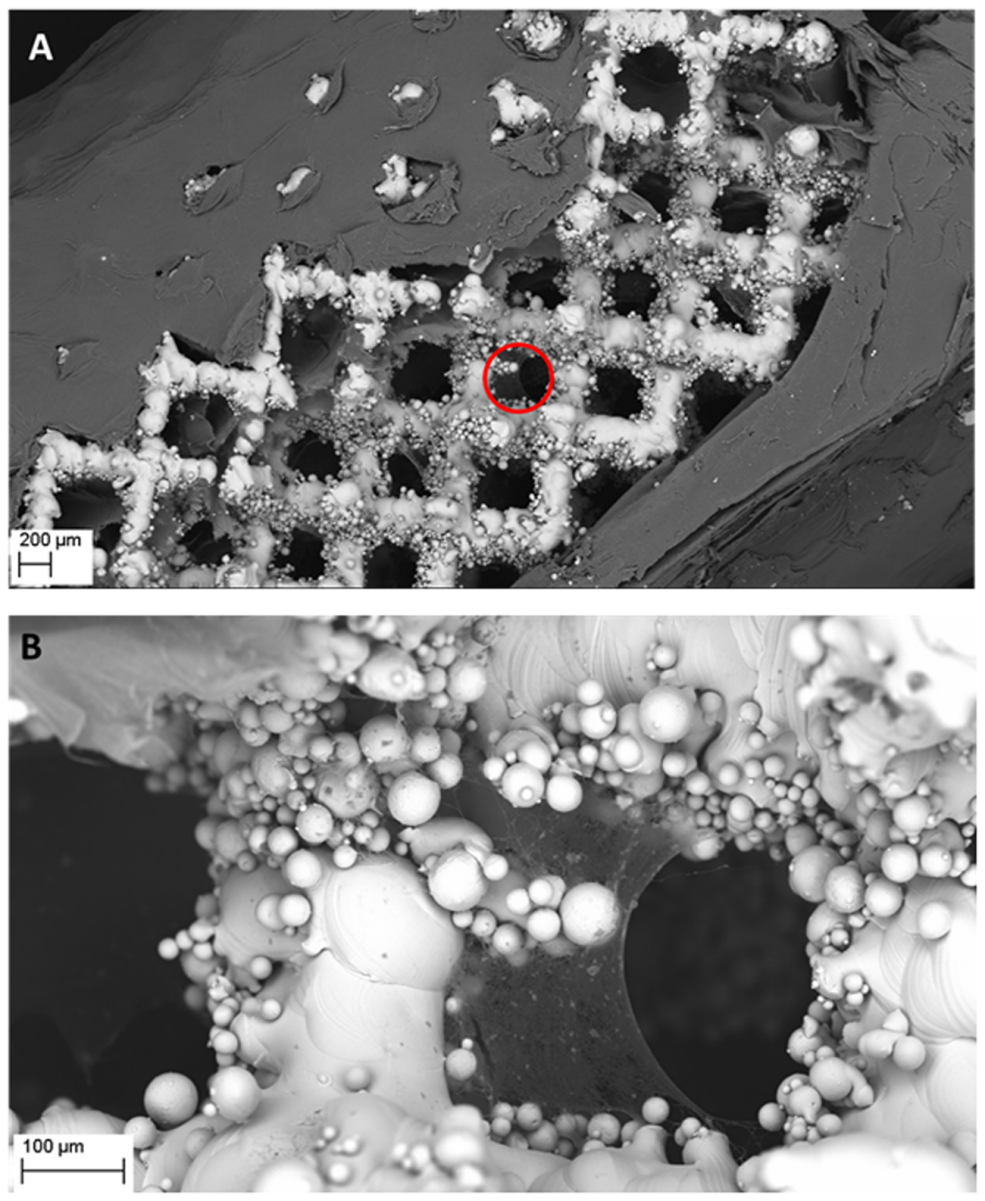
Visualization of cellulose covering (A) and ingrowing (B) the pores of Ti6Al7Nb implant. The area marked by a red circle in (A) is visualized with higher magnification in (B). The cellulose seen in (A) was intentionally mechanically disrupted (as seen in the middle of the picture) to uncover the underlying implant’s struts. Magn. 51x and 318, respectively. Zeiss EVO MA SEM Microscope. Please also refer to S3 Fig to see more Ti6Al7Nb implants with partially removed cellulose.

The structure of cellulose coating the implants visualized by SEM is presented in Fig 2. It should be emphasized that as we have proven in our earlier work, the presence of un-sintered metal powder (as seen in Fig 2B) does not affect the mechanical properties of the implants manufactured [30,31]. Additional data concerning the chemical composition of the manufactured scaffolds are presented in S1 Table. For the reader’s convenience we have also depicted the process of cellulose accretion within bigger implants of cubical form (S2 Fig).

Having proven the ability of BC to coat the implants, we analyzed the features of such modified structures that are crucial in the context of orthopedic applications.

**Fig 3:**
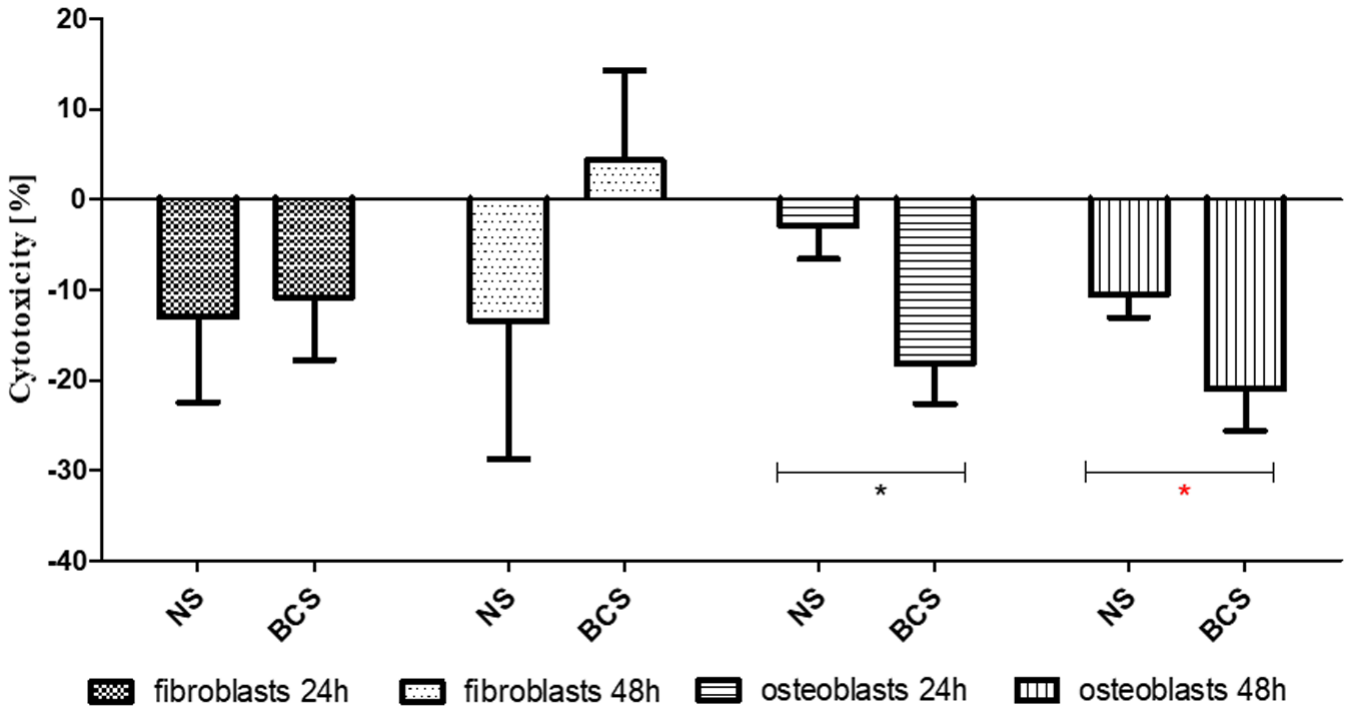
Cytotoxicity of BC-coated Ti6Al7Nb scaffolds vs. non-coated Ti6Al7Nb native scaffolds for fibroblasts and osteoblasts after 24 and 48 h of incubation. BCS—BC-coated Ti6Al7Nb scaffolds; NS—non-coated Ti6Al7Nb native scaffolds; Asterisks mark statistically significant differences (M-W test, p<0.5) between particular columns.

**Fig 4:**
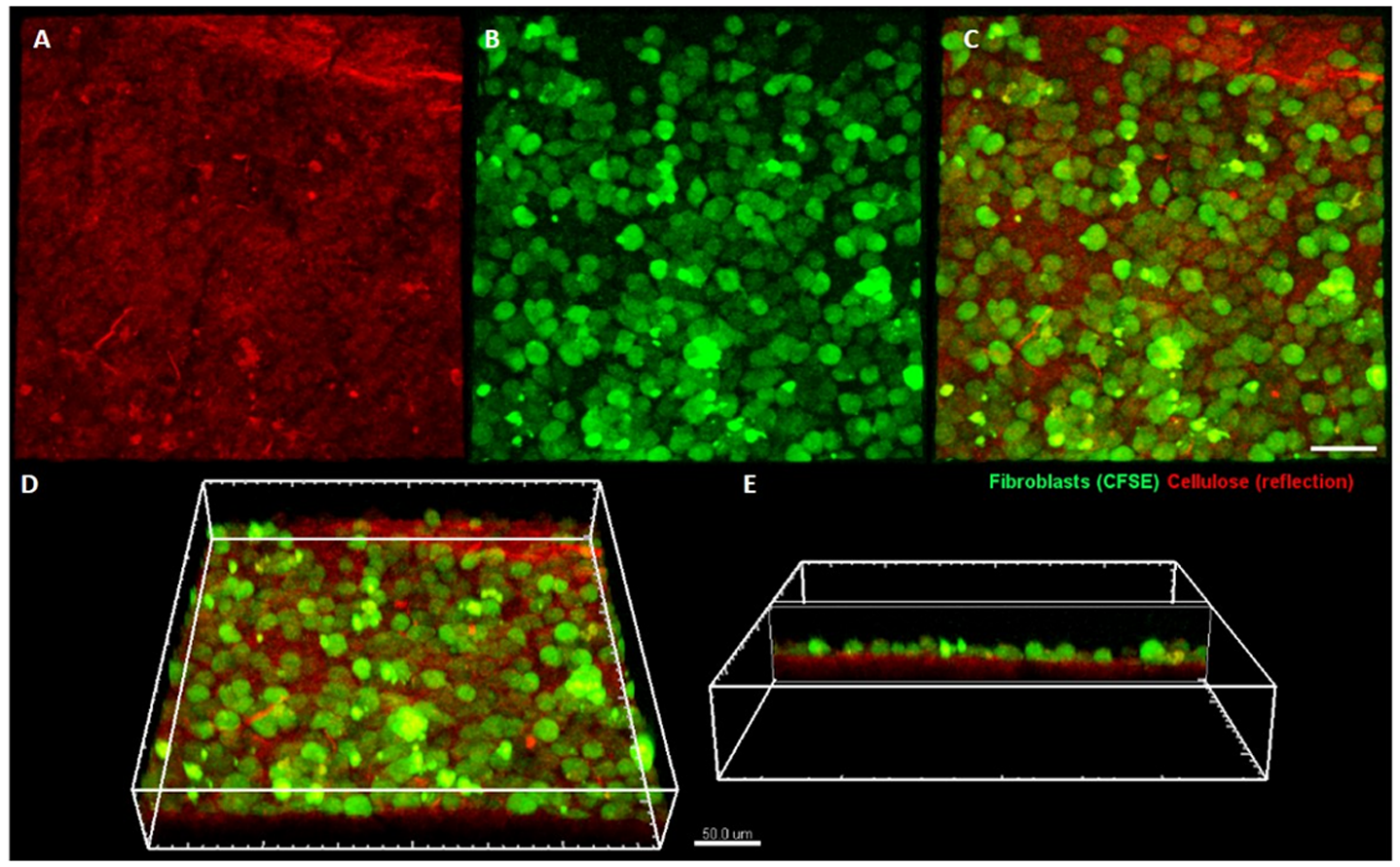
Fibroblasts colonizing bacterial cellulose membrane. Cellulose is visualized using laser reflection (shown in red) (A), while fibroblasts are fluorescently labeled (shown in green) (B). Merged channels are presented in (C) (view from the top), (D) and (E) (side views). The imaging was performed on a Leica SP8 confocal microscope.

As shown in Fig 3, BC-coated scaffolds displayed no cytotoxicity against fibro- and osteoblast cell lines according to a binding standard. It is worth noting that the number of osteoblasts was significantly higher when incubated with extracts obtained from the BC-coated implants in comparison to implants devoid of BC surface, regardless of the time of incubation (M-W test, p<0.05). Moreover, the cellulose membrane supported the growth of fibroblasts which densely covered its surface and formed a cell monolayer, as visualized with confocal microscopy (Fig 4).

The above-presented findings regarding high biocompatibility and very low cytotoxicity of implants modified with cellulose stay in line with the reports of other research teams which showed that BC is an excellent environment for cell growth and development [13,29,32].

It should be emphasized here that native BC does not show any antimicrobial properties. However, its high ability to absorb fluids allows it to be easily saturated with a wide spectrum of substances, including antimicrobial agents. The microfibrillar structure of BC is responsible for the prolonged time of drug release and provides long-lasting antimicrobial protection, what is an extremely desired feature in the context of orthopedic infections. Therefore, we saturated the BC-coated implants with a spectrum of gentamycin concentrations and tested them against the main etiological factor of bone infections, i.e. *S*. *aureus* [33,34]. This particular *S*. *aureus* strain was gentamycin-sensitive. Its MIC, measured by standard micro-dilution method, was 1.95 μg/mL.

The introduction of BC-modified, gentamycin-saturated implants onto *S*. *aureus* cultures led to a noticeable decrease in the above bacteria’s growth (expressed as an inhibition of growth zone around the implant) as presented in Table 1. It is worth noting that the inhibition zones for gentamycin concentrations were higher for BC-coated than for non-coated Ti6Al7Nb implants.

**Table 1:** Inhibition zones of *S*. *aureus* with regard to type of implant and antibiotic concentration applied.

		Gentamycin concentration applied [mg/mL]					
		1.0	10.0	8.0	4.0	2.0	0.0
**Growth inhibition zone [mm]**	BCS	32.0	23.0	20.0	16.0	16.0	0.0
	NS	30.0	16.0	18.0	13.7	11.0	0.0

BCS—BC coated; NS—BC non-coated.

**Fig 5:**
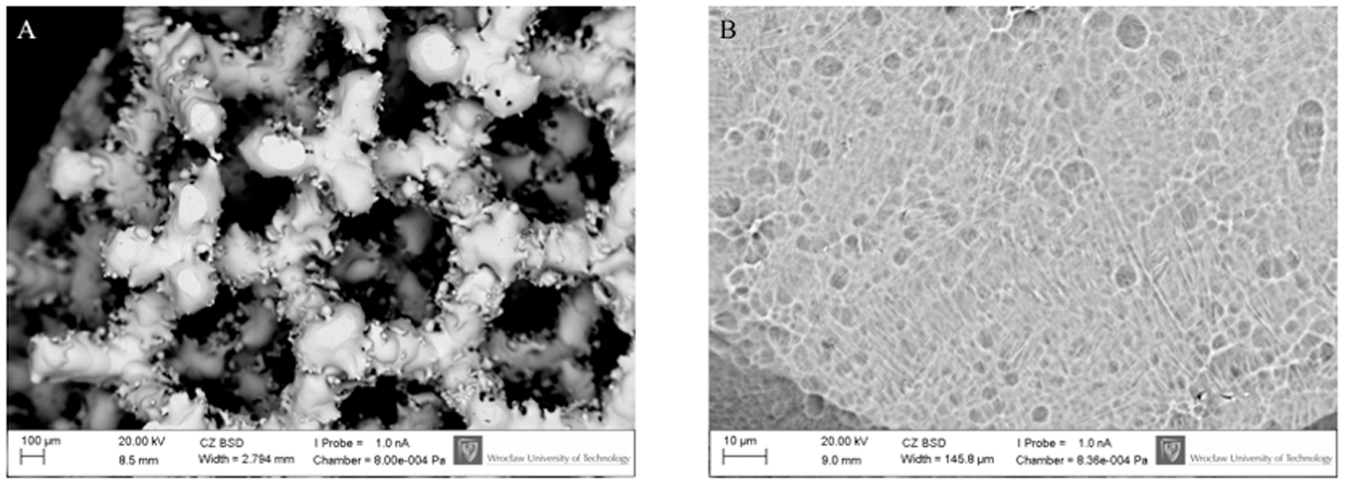
No negative impact of the procedures applied on implant structure. Structure of the implants coated with cellulose and subjected to a temperature of 80°C (after cellulose removal) under (A) 105x and (B) 2060x magnification, respectively. Zeiss EVO MA SEM Microscope.

To the best of our knowledge, we are the first research team to have used live bacteria to directly coat an implant with biopolymer surface. The cultivation of implants with bacteria and the obvious necessity of subsequent removal of *K*. *xylinus* cells from BC drove us to perform the last stage of this line of investigation, namely to check the potential impact of culturing, alkaline purification including high temperature (80°C) on the implant structure. As can be seen in Fig 5, no differences between BC-modified vs. non-modified implants were observed. It also stays in line with the reports of other teams, which showed that it is not alkali lysis but rather the use of hydrofluoric and nitric acid that may lead to the alteration of the titanium surface due to this element’s affinity to form hexafluorocomplexes [35]. For the reader’s convenience we have also performed a similar analysis using digital microscopy approach toward bigger implants of cubical form (S5 Fig). Likewise, no alteration of structure was observed.

In the course of implant-related infection, a bone or joint damage may be aggravated due to tissue degeneration and inflammation. If improperly treated, a local infection may turn into a systemic one, putting the patient’s life in danger [23,34]. Therefore, antimicrobial prophylaxis and quick implant incorporation to the bone is of pivotal significance with regard to the patient’s safety. Herein, we have experimentally demonstrated that Ti6Al7Nb implants coated with BC and saturated with gentamicin have antimicrobial activity against *S*. *aureus* and display high biocompatibility with regard to fibroblasts and osteoblasts. Further investigation on bigger size implants is undoubtedly needed but the data presented here provides strong arguments for a future use of BC-modified implants in clinical practice.

## Conclusions

Implants coated with BC and saturated with gentamycin display very low cytotoxicity and are able to stop proliferation of bone pathogen, *S*. *aureus*. Moreover, the method of implant coating applied and pioneered by us does not flaw the biomaterial surface. Therefore, the coating method presented here meets the demands of modern implantology and is of high suitability in orthopedic applications.

## Supporting information

S1 Fig CAD model of cylindrical scaffold used in the presented study (A) and CAD model of bigger, cubical scaffold used to better visualize cellulose layers growing inside of pores (B). (TIFF)

S2 Fig Stages of covering Ti6Al4V implant with BC. A)—native cubical Ti6Al4V implant; B)—initial stage of coating implant by BC; C)—cubical implant partially covered with BC; D)—implant covered with BC (BC is visible also inside the implant pores (red arrow), please refer to Fig 2 of main body of the manuscript to see ingrowth of cellulose within cylindrical implant visualized by SEM technique); E)—BC-coated implant after removal from culturing plate. The yellow color of BC is caused by the presence of bacteria and their media leftovers; F)—BC-covered implant after chemical purification.
(TIFF)

S3 Fig Ti6Al7Nb cylindrical implants covered with BC, partially digested with cellulase enzyme. Magn 100x, 250x, 450x, 2060x for pictures: A,B,C,D, respectively. Zeiss EVO MA SEM Microscope.
(TIFF)

S4 Fig A,B—Intact Ti6Al4V implant structure; C,D—Ti6Al4V cubic implant structure after coating, cleansing and removal of BC. Pictures taken using digital microscope (Keyence VR-3000).
(TIFF)

S1 Table Chemical composition of the manufactured scaffolds. (DOCX)
