# Supplementary figures and images for: Development and biological evaluation of Ti6Al7Nb scaffold implants coated with gentamycin-saturated bacterial cellulose biomaterial
Source: PLoS One. 2018 Oct 24;13(10):e0205205. doi: 10.1371/journal.pone.0205205 (PMC6200220; doi:10.1371/journal.pone.0205205)

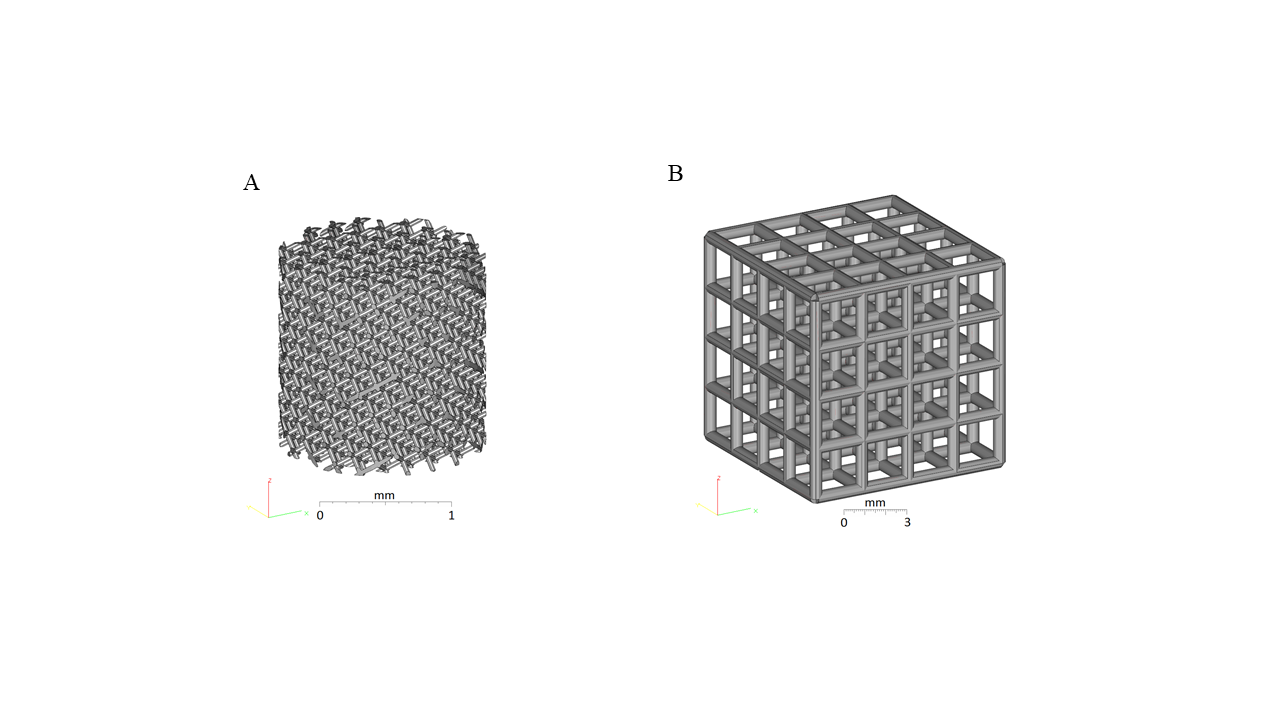

Supplement: S1 Fig — (TIFF) [file pone.0205205.s001.tiff]

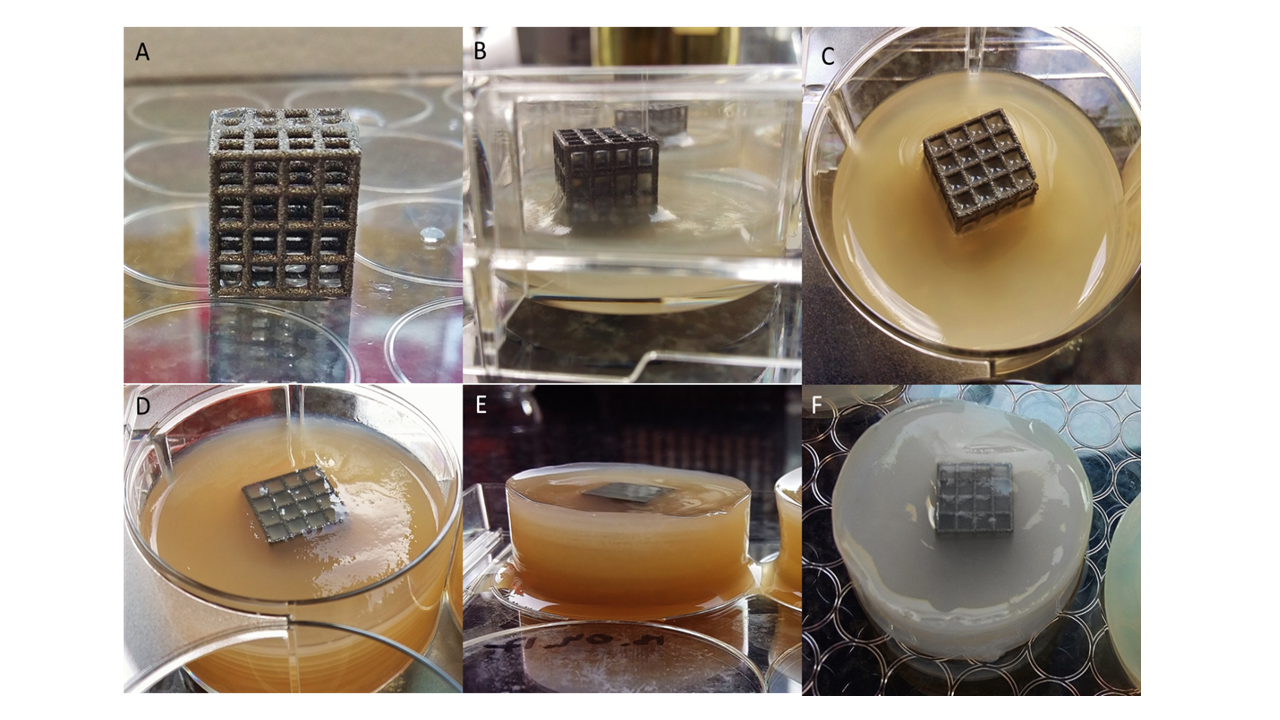

Supplement: S2 Fig — A)—native cubical Ti6Al4V implant; B)—initial stage of coating implant by BC; C)—cubical implant partially covered with BC; D)—implant covered with BC (BC is visible also inside the implant pores (red arrow), please refer to Fig 2 of main body of the manuscript to see ingrowth of cellulose within cylindrical implant visualized by SEM technique); E)—BC-coated implant after removal from culturing plate. The yellow color of BC is caused by the presence of bacteria and their media leftovers; F)—BC-covered implant after chemical purification. (TIFF) [file pone.0205205.s002.tiff]

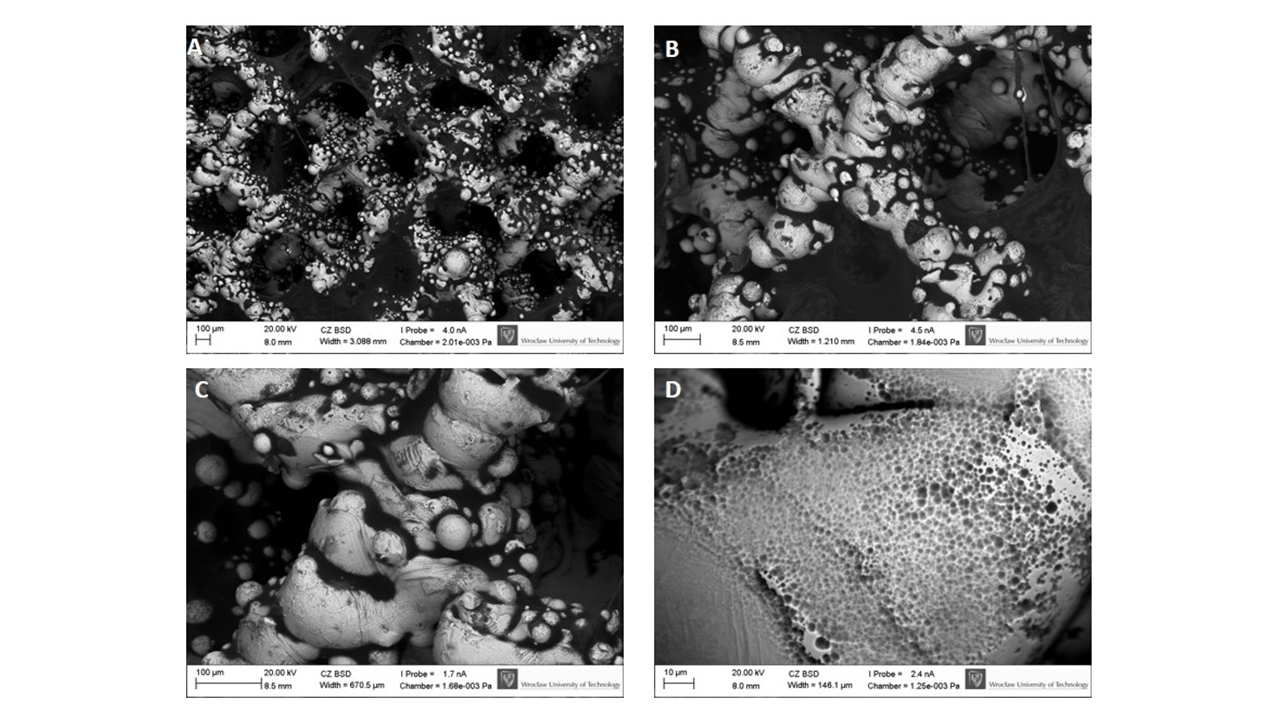

Supplement: S3 Fig — Magn 100x, 250x, 450x, 2060x for pictures: A,B,C,D, respectively. Zeiss EVO MA SEM Microscope. (TIFF) [file pone.0205205.s003.tiff]

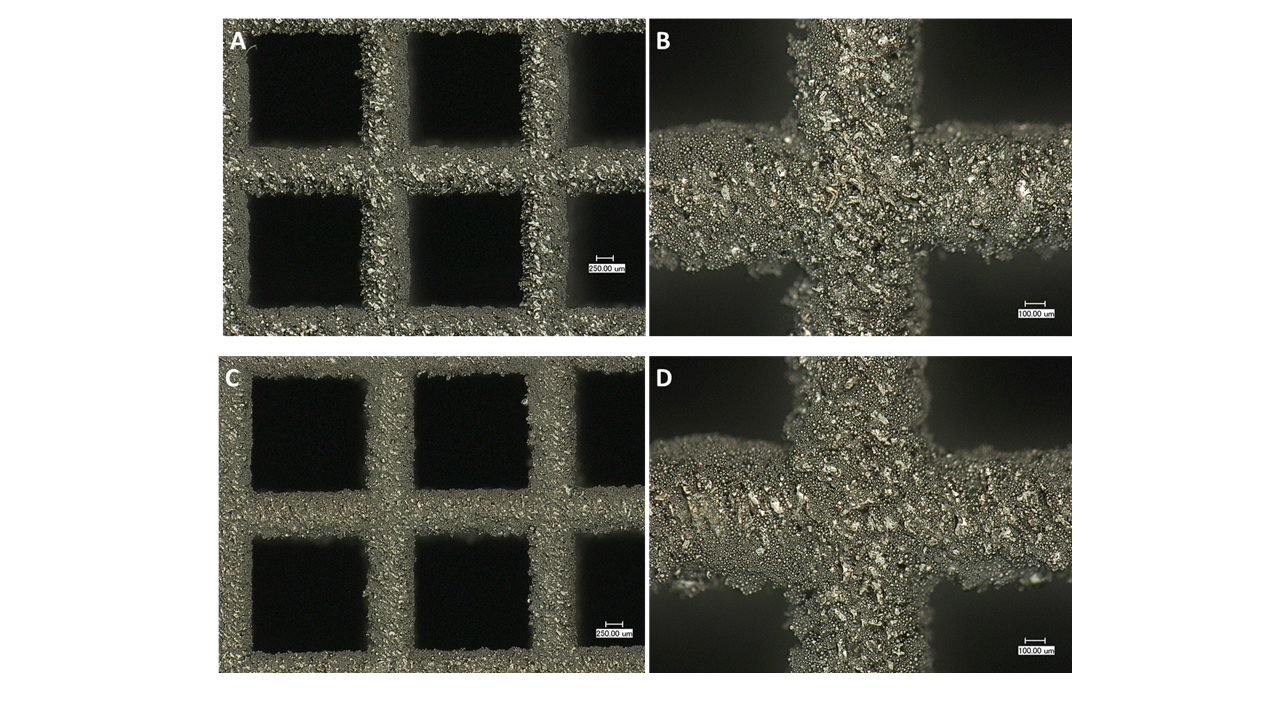

Supplement: S4 Fig — Pictures taken using digital microscope (Keyence VR-3000). (TIFF) [file pone.0205205.s004.tiff]
