# Supplementary material for: Development and biological evaluation of Ti6Al7Nb scaffold implants coated with gentamycin-saturated bacterial cellulose biomaterial
Source: PLoS One. 2018 Oct 24;13(10):e0205205. doi: 10.1371/journal.pone.0205205 (PMC6200220; doi:10.1371/journal.pone.0205205)
Supplement: S1 Table — (DOCX) [file pone.0205205.s005.docx]

**S1 Table. Chemical composition of the manufactured scaffolds.**

| **Element** | **Mass content [%]** | |
| --- | --- | --- |
|  | **Sample** | **ASTM F1295-11 standard^a^** |
| C | 0.017 | 0.08 – 0.10 |
| H | 0.0046 | 0.009 – 0.011 |
| N | 0.049 | 0.05 – 0.07 |
| 0 | 0.21 | 0.20 – 0.22 |

^a^ - acceptable content according to ASTM F1295-11 standard. Point 7 of this standard covers the chemical requirements and product analysis tolerances for wrought annealed, cold-worked or hot-worked Ti6Al7Nb alloy (in form of bar, wire, sheet, strip, and plate) to be used in the manufacture of surgical implants.
